# The Effectiveness of an App-Based Fitness Program on Self-Perceived Physical Functioning in Older Adults: Randomized Waitlist-Controlled Trial

**DOI:** 10.2196/64922

**Published:** 2025-08-18

**Authors:** Siegfried Eisenberg, Birgit Trukeschitz

**Affiliations:** 1 Institute for Advanced Studies, Health Economics and Health Policy, Vienna, Austria; 2 Research Institute for Economics of Aging, WU Vienna University of Economics and Business, Welthandelsplatz 1, Vienna, 1020, Austria, 43 1313365877

**Keywords:** physical functioning, fitness app, ICT, randomized waitlist-controlled trial, dependency, fitness program, mobile health, app, older adults, physical activity, information and communications technology

## Abstract

**Background:**

A decline in physical functioning can result in a loss of independence, particularly in older adults. Information and communications technologies supporting physical activity, such as fitness apps, are perceived as promising tools to increase activity levels. However, only little is known about fitness apps’ impact on older people’s abilities and skills to accomplish activities of daily living.

**Objective:**

In this study, we aimed to investigate whether a newly developed app-based physical activity program improves self-perceived physical functioning or at least prevents a functional decline in older adults.

**Methods:**

We targeted older adults in their early years of retirement and conducted a randomized waitlist-controlled trial in Austria. The app-based program was received by the intervention group (IG) for a period of 14 weeks first; afterwards, the IG handed over the devices to the control group (CG). Both groups had 3 appointments with a fitness coach. The app comprised 3 functions, a multicomponent fitness exercise program, recommendations for outdoor activities, and e-learning courses. Self-perceived physical functioning was measured by 4 common daily life activities rated on 6-point scales. Data were collected through online surveys at 3 time points, 8 weeks before intervention start (t_-1_), at intervention start (t_0_), and 14 weeks later at the end of the intervention (t_1_). We estimated generalized linear mixed models and derived average marginal effects. The effects are presented as differences in percentage points resulting from differences in estimated probabilities between groups before and after the intervention.

**Results:**

A total of 219 participants between 60 and 72 years, 96 in the IG and 123 in the CG, were analyzed. The intervention significantly increased the self-perceived abilities of “climb up stairs and carry something” (odds ratio [OR] 2.67, 95% CI 1.37-5.18; *P*=.004) and of “lift and carry groceries” (OR 1.99, 95% CI 1.02-3.89; *P*=.04). On the contrary, no significant impact on the ability to “walk 1 km” (OR 1.91, 95% CI 0.85-4.30; *P*=.12) and “stretch to the toes” (OR 1.31, 95% CI 0.62-2.76; *P*=.48) was found. The probability of rating “climb up stairs and carry something” as “very easy” increased by 8.8 percentage points (95% CI 2.6-14.9; *P*=.005) and “lift and carry groceries” by 7.9 percentage points (95% CI 0.5-15.3; *P*=.04). Predicted probabilities showed that outcomes improved in the IG and remained unchanged in the CG.

**Conclusions:**

Although the fitness app was designed as a multicomponent program, it supported only selected capabilities relevant for independent living. The app-based physical activity program increased self-perceived physical functioning related to strength, endurance, and balance, but not to flexibility. This highlights a clear need for future apps and research to focus on all relevant areas, including flexibility and mobility, which are crucial for fully maintaining independence in older adults.

## Introduction

Physical functioning is key for independent living and affects quality of life in older adults [1,2], as well as personal networks and health care systems [3]. Mobility and autonomy are among the most important domains determining quality of life in older adults [4]. Independent older adults require less support by kin and nonkin. Thus, family and other members of the personal network might be exposed to less mental stress and need less financial and time efforts, for example, for informal care and caring costs. As health care systems face challenges in terms of increasing health care costs and decreasing workforce in aging populations [5], longer periods of independent living would contribute to better reallocating scarce resources to those needing them most. Thus, physical functioning is relevant for independent living and support “successful aging” and “aging in place.”

“Successful aging” [6,7] and “aging in place” [8,9] build on the maintenance of physical independence. “Successful aging” focuses on the quality of aging and comprises several dimensions, whereby a distinction is made between biomedical aspects (eg, physical functioning) and psychosocial factors (eg, actively engaged in life) [10]. These aspects and factors also describe conditions for “aging in place” that, generally, refer to the objective of remaining in one’s own home and maintaining some level of independence and autonomy [8]. Both concepts stress the importance of physical functioning that was found to decline faster than psychological and social well-being [11].

In the last decade, innovations in information and communications technology (ICT) have opened new opportunities to support “successful aging” and “aging in place.” ICT-supported physical activity programs were effective in increasing physical activity levels [3,9,12,13] and are known for their impact on physical independence at an early stage of aging [14]. Compared with non–ICT-based interventions, ICT-based interventions have several advantages. They can be easily accessed at any time and place and more conveniently integrated into daily life routine [15]. In addition, with comparatively low effort, an unlimited number of people can be reached [16]. Thus, ICT is a promising way to deliver physical activity programs to a broad range of community-dwelling older adults, as supported by reviews [17-20] and were also effective in preventing physical health declines in older adults during isolation periods [21].

So far, only a few studies have investigated the impact of an ICT-based intervention directly on physical functioning in older adults, and less have considered self-perceived physical functioning and its implication for coping with situations in daily life. Studies reported significant improvements in strength, endurance, and balance [22], skeletal muscle mass [23], gait [24], and strength and flexibility [25]. Hence, this evidence referred to objectively measured domains of physical functioning (eg, the 30 seconds Chair-Rise Test). Also, the impact of the app-based fitness program assessed in this paper was already evaluated using objectively measured physical functioning in previous research, showing a significant improvement in strength and flexibility for a subsample of female participants [25]. The restriction to female participants resulted from a lack of objectively measured data. However, there are hardly any insights into the impact of these technologies on how well users perceive themselves to be able to cope with common everyday activities [26-29].

In the context of daily life situations, solely objectively measured physical functioning might not be able to predict whether older adults perceive themselves as able to carry out activities, as the belief in one’s own ability reflects the confidence to perform certain activities, also denoted as self-efficacy [30]. Self-efficacy is the main construct of the social cognitive theory and is decisive whether people engage in activities or not [31]. Studies show that self-efficacy affects physical activity behavior and is mentioned to facilitate the maintenance of fitness exercises [32-34]. In particular, ICT-based interventions offer the opportunity to address self-efficacy by promoting the health benefits of physical activity (eg, via SMS text message or direct prompts in the app), additional to a physical activity program [35]. At the same time, self-efficacy can be increased by physical activity and is expected to improve physical performance in older adults [33]. The investigation of self-perceived physical functioning includes self-efficacy beliefs. Thus, studies investigating the impact of ICT-based physical activity programs on self-perceived physical functioning and, consequently, physical independence in daily life situations in older adults are needed but still missing.

In this study, we thus investigated the impact of a newly developed app-based physical activity program on self-perceived physical functioning in older adults in their early years of retirement. This group of older adults was expected to benefit largely from the preventive character of the intervention on functional decline and to be at least somewhat tech-savvy, which facilitates operating apps. As the intervention addressed self-efficacy by tailoring the app-based program to the needs and expectations of the target group [36], we assessed the effectiveness of the fitness app on the users’ self-perceived abilities to perform selected activities of daily living. People’s perceptions of changes in their performance may support them in taking control of their health and fitness.

## Methods

### Framework for the Analysis and Hypotheses

**Figure 1.**
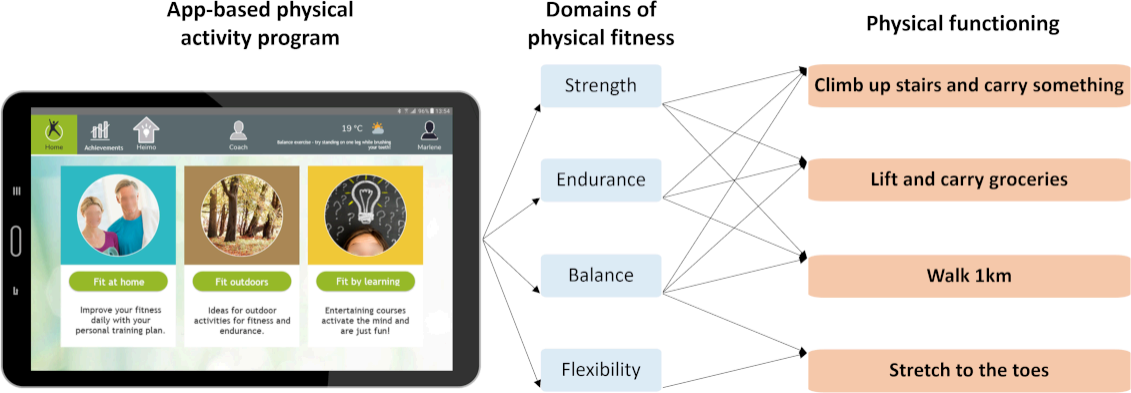
Impact model for the app-based physical activity program on physical functioning (Source: Screenshot - ILSE App, M.I.T e-Solutions GmbH, Reprinted with permission and faces blurred for publication; conceptual model by the authors).

Figure 1 illustrates the rationale for the expected impact of the ICT-based physical activity program on self-perceived physical functioning in daily life situations adapted from Jones and Rikli [37]. The multicomponent app-based physical activity program was developed to improve domains of physical fitness which can support users in conducting activities of daily living. The outcomes measured thus represent common daily activities that are important for independent living [28]. “Climb up stairs and carry something” and “lift and carry groceries” are common activities that require strength, endurance, and balance. “Walk 1 km” requires strength, endurance, and balance. “Stretch to the toes” (eg, to tie shoelaces) requires balance and flexibility.

To expect any effects, a reasonable take-up of a program is required. The take-up of the newly developed multicomponent app-based fitness program was reported to be good, with 70% (37/52) of the participants using the app-based program at least once a week and 25% (13/52) using it 4 times per week or more often [25,30]. We thus expected the app-based program to affect self-perceived physical functioning in daily life situations. It was therefore hypothesized that if the app-based fitness program supports older people’s physical functioning, the intervention will be reflected in participants’ perceptions to increased or prevented decline in abilities to “climb up stairs and carry something” (hypothesis 1), “to lift and carry groceries” (hypothesis 2), to “walk 1 km” (hypothesis 3), and to “stretch to the toes” (hypothesis 4).

### Study Design

We conducted a randomized waitlist-controlled trial with 2 groups, an intervention group (IG) and a control group (CG) in 2 Austrian provinces, Vienna and Salzburg. The IG received the newly developed fitness app provided on a tablet and a 3D camera and a fitness tracker paired with the app. The IG had 3 individual meetings with a fitness coach for a period of 14 weeks. The CG was put on a waitlist, which means they did not receive any technology at the same time as the IG but also had 3 individual meetings with a fitness coach. For both groups, these appointments aimed to assess objectively measured physical functioning and for the IG to distribute and explain the technological equipment. After the IG had completed the intervention period, the CG participants were equipped with the app-based fitness program and had access to another 3 appointments with their fitness coach. As both groups had 3 appointments with a fitness coach in the main trial period, this design allowed us to single out the effect of the fitness app component.

The fitness app program was developed for older adults in their early years of retirement. We targeted pensioners in 2 Austrian provinces who had been receiving old age pension between 3 and 6 years. On behalf of the research team, the Austrian Social Pension Insurance Agency sent out invitation letters to 10,000 randomly selected pensioners in the targeted population in 2018. The letters contained information on the research project, the app-based physical activity program, and an instruction on how to enroll for the free trial period of the program. Eligibility was assessed after enrollment to the program. People enrolling in this program were excluded if they reported severe functional limitations or disability (eg, using a wheelchair) or were already very active (eg, physical activities at least 5 times a week) or already had a fitness coach.

Using Stata 15’s (StataCorp) [38] random number function, we randomly allocated subscribers meeting all criteria to the IG and CG and sent information emails to participants in both groups. CG participants were informed that they were put on a waiting list to use the app-based fitness program about 6 months later but to already be eligible to meet the fitness coach. The CONSORT-EHEALTH (Consolidated Standards of Reporting Trials of Electronic and Mobile Health Applications and Online Telehealth) checklist is reported in Checklist 1.

### Data Collection

For the effectiveness analysis, we collected data using a 1-time and a repeated questionnaire. All questionnaires were created with LimeSurvey 2.73 (Carsten Schmitz & LimeSurvey Team) [39]. The 1-time questionnaire was implemented in the registration form for the project available on the website. The registration form was only accessible from October to December 2018 by using the code on the invitation letter. Data from this questionnaire were used to assess eligibility, for sample description, and as covariates of the models.

Repeated questionnaires were used to measure the impact of the intervention on certain outcomes. It was sent to participants at 3 time points, in February 2019 after the allocation into IG and CG (t_–1_), 8 weeks later at the intervention start (t_0_), and 14 weeks later at the end of the intervention (t_1_). The questionnaires had to be completed before meeting the fitness coach. Participants who did not complete the trial were asked to fill in the questionnaires to enable an intention-to-treat analysis.

An approximated a priori sample size calculation using GPower v3.1.9.2 [40] for a comparison of 2 groups with 7 covariates resulted in a total sample size of at least 210 participants to achieve a power of 95% with an error type 1 probability of 0.05 for a medium effect size of 0.25 [41].

### Intervention Characteristics

The intervention comprised a newly developed fitness app provided on 2 devices, a tablet (Samsung Galaxy Tab A) and a 3D camera (Orbbec Persee) that could be mounted on a television or PC monitor and an activity tracker (Samsung Gear Fit 2 Pro) paired with the app but with no further modifications. We used hardware components available on the market and developed new software to adjust the app-based fitness program to the needs of the target group. The development of the app followed a user-centered design approach, by involving 12 key users in the age group of the target population [36]. In addition, we followed the recommendations by Chase [42] and Harrington et al [43] on the development of ICT-based physical activity interventions for older adults to ensure that designs, such as font size and functions, were adequate for the target group. Usability and user experience were assessed after the trial and showed that, in general, most participants rated their tech-savviness as moderate and about 80% (64/78) of the participants mentioned that learning to use the app requires low effort and the app is easy to use [44].

**Figure 2.**
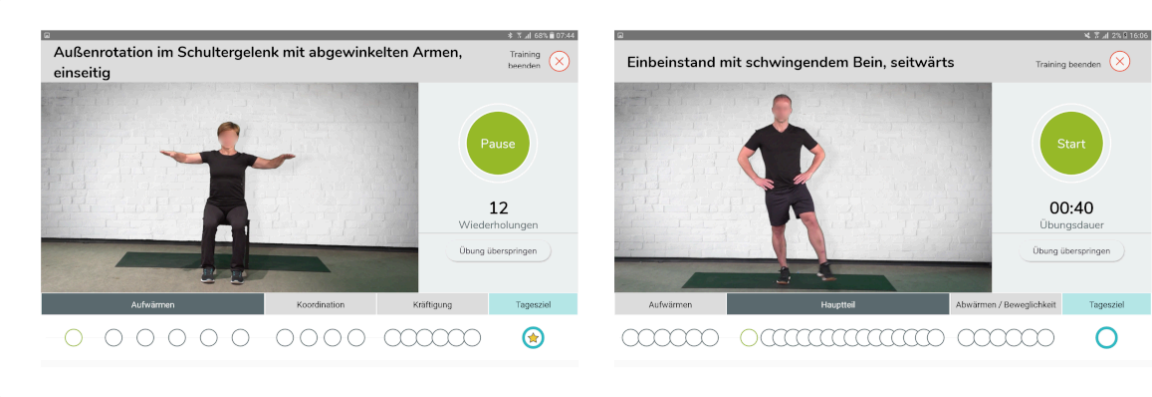
Fitness app user interface of exercise program (2 sample exercises) (Source: Screenshots of the fitness app, © M.I.T e-Solutions GmbH, Reprinted with permission and faces blurred for publication).

The fitness app comprised 3 functions tailored to the needs of older adults and was provided on a tablet for all participants in the IG. The first and main function was a new multicomponent fitness exercise program developed by sport scientists with daily changing fitness exercises customized to people older than 60 years. As Figure 2 illustrates, fitness exercises were demonstrated in short video clips supplemented by written explanations. Video models were chosen from the target group to enhance self-efficacy beliefs. The name of the exercise was displayed on the top of the screen, while the progress of a session was displayed underneath the video. Progress circles represented exercises divided into 3 phases denoted as “warm-up,” “main part,” and “cooldown.” Accordingly, the screenshot in Figure 2 on the left-hand side shows a “warm-up” exercise, which has to be repeated 12 times, the screenshot on the right-hand side represents an exercise of the main part, which has to be conducted for a duration of 40 seconds. The 3 phases addressed different physical abilities. Exercises in the warm-up section (eg, marching in place) referred to endurance. The main part consisted of mixed exercises for strength (eg, chair squats) and balance (eg, uni-pedal stance), and exercises in the cooldown section addressed flexibility (eg, hip flexor stretch). Participants received fitness exercises according to their fitness level assessed by a fitness coach at the 3 points in time. Within their level, each participant could choose between a 10-, 20-, and 30-minute exercise session. An in-depth explanation of the whole fitness program was published by Jungreitmayr et al [25].

The second function of the app-based fitness program offered access to an implemented well-known app for outdoor activities available in app stores [45]. The user interface was modified to allow a more convenient search for walking or cycling suggestions on tablets. The third function offered more than 20 e-learning courses with fitness-related topics referring to physical activities, motivation, stress management, and relaxation. This function was implemented to highlight the importance of physical activity, to improve self-efficacy, and to give further recommendations for a healthy lifestyle. In addition to these functions, the app featured an activity summary (eg, the number of completed fitness exercises, steps, and e-learning courses) on a daily and weekly basis and included weekly rewards as virtual trophies.

### Measures

#### Outcome Measures

Self-perceived physical functioning was measured by 3 items taken from the composite physical function (CPF) scale by Rikli and Jones [28]. The CPF scale measures the ability to perform common daily life activities to maintain independence. We selected the following items of the CPF scale as most appropriate to assess an impact of the multicomponent fitness exercises program on our target group: “climb up and down a flight of stairs,” “lift and carry 10 pounds,” and “walk 1/2 mile.” The rationale was to choose items that reflect some difficulty, since the participants were expected to be rather independent. We translated questions and response options into German and validated them through cognitive interviews [46] with key users. The results of cognitive interviews suggested slightly changing the wording to increase the appropriateness of the items for the target group and to add examples to enable a better imagination of the activity. Hence, for our target group, we used “climb up stairs and carry something” (refer to hypothesis 1) and asked respondents to imagine carrying a full laundry basket or a box of empty bottles, “lift and carry groceries” (refer to hypothesis 2) was supported by the hint to imagine carrying a full bag of groceries for about 250 meters and “walk 1 km” (refer to hypothesis 3) with the hint that this equals a walk of about 15 minutes. To measure abilities of daily living that require flexibility, we selected “stretch to the toes” (refer to hypothesis 4) and asked respondents to imagine tying the shoelaces. In addition, this item served as a subjective equivalent to the Toe-Touch Test, which is known for its assessment of flexibility [47].

All 4 items were measured on a 6-point scale to fine-grade the response options and to enable measuring changes at a lower level compared with the original scale that offers only 3 response options, indicating the level of help needed. As our target group was much more capable, we used a scale to capture the degree of difficulty of performing the tasks. The scale selected ranged from “very difficult” (1) to “very easy” (6), while outcome levels between end points were not labeled, also known as end labeling with numerical values. In addition, we offered the option “I don’t do that,” which was considered as missing value in the analyses. We did not offer a neutral response option to encourage respondents to decide whether this task is (very or somewhat) easy or (very or somewhat) difficult for them. To facilitate interpretation of the results, the six outcome levels were labeled as follows: (1) “very difficult,” (2) “difficult,” (3) “somewhat difficult,” (4) “somewhat easy,” (5) “easy,” and (6) “very easy.”

#### Covariates

We adjusted the estimated effect size by age, sex, education, health, and previous physical activity, since these factors are known to potentially affect physical activity behavior [48-53]. We used age as a metric variable and coded men “0” and women “1.” We classified education on 5 levels based on the International Standard Classification of Education 2011 (ISCED 2011): “up to lower secondary education” (ISCED 0 to 2) (0), “upper secondary education” (ISCED 3) (1), “post-secondary education” (ISCED 4) (2), “short-cycle tertiary education” (ISCED 5) (3), and “bachelor’s degree or higher” (ISCED 6 to 8) (4). Self-rated health status was measured by the German translation of the 36-Item Short Form Health Survey [54] on a 6-point scale ranging from 0, the worst, to 5, the best possible health status and treated as metric. Participants were asked to report their number of physical activities at the registration questionnaire on the following scale, “never” (0), “less than once a week” (1), “1‐2 times a week” (2), and “3‐4 times a week” (3). In addition, we included a dummy for the 2 Austrian provinces, with “0” for Vienna and “1” for Salzburg. Participants were asked to inform about their household size by integer numbers. Self-rated health status was the only time-varying covariate; all other covariates were time-invariant.

### Statistical Analysis

To estimate the intention-to-treat effect, we estimated ordinal mixed logit models with individual-specific random intercepts to account for repeated responses by participants. In these models, the treatment effect of the ICT-based intervention on physical functioning outcomes is estimated by including an interaction term of a dummy variable that indicates the IG (1) and CG (0) and a dummy variable for period of data collection, that is, before (0) and after (1) the intervention. This method considers preintervention differences that are, generally, assumed to be random within a randomized controlled trial [55], but nevertheless, might bias the estimated effect size [56]. Thus, we additionally added covariates capturing sociodemographic differences and health and physical activity levels at enrollment to our model to increase precision of estimates [57].

**Figure 3.**
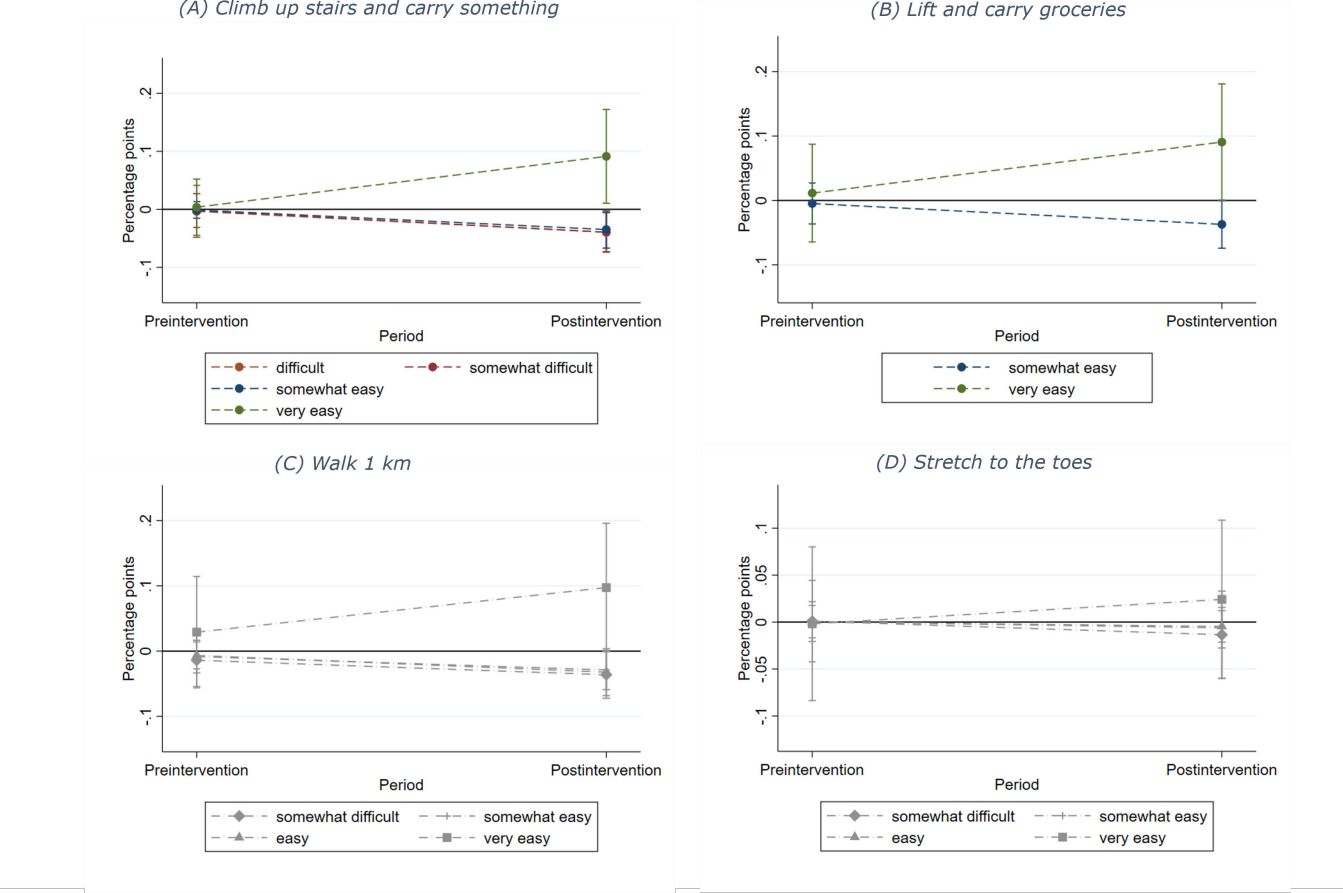
Average marginal effects for each outcome between IG and CG (Source: own calculations). Note: error bars represent 95% CIs in all figures; in (A) and (B) statistically nonsignificant outcome levels are not shown for better visualization of the significant effects and in (C) and (D) statistically nonsignificant outcome levels are represented in gray and symbols other than circles.

According to the nature of ordinal outcome data and estimated models, we derived average marginal effects to report on the differences in probabilities between groups and time. The results are presented as percentage points indicating whether an outcome level is expected to increase or decrease [58]. These average marginal effects are illustrated in Figure 3D. Estimation results denoted as “pre” represent the estimated average probability of both preintervention periods without intervention (t_–1_ and t_0_) and “post” refers to the estimated probability after the intervention (t_1_).

To investigate the origin of the effect—if effects resulted from an increase in the IG or from a decline in the CG—we present predicted probabilities for each outcome level before and after the intervention for the outcome levels. A significant difference between groups is interpreted as a change in an ability to perform specific activities of daily living and, consequently, as an impact on physical functioning.

Before our analyses, we conducted Little’s Missing Completely at Random test to examine missing data [59]. Test statistics confirm that missing values can be treated as missing completely at random. Furthermore, we collapsed outcome levels with a low number of observations into an adjoining category to reduce Type I error probability [60]. We used Stata 17 [61] for all calculations and considered a *P* value lower than .05 as statistically significant.

### Ethical Considerations

The study design was approved by the Ethics Commission in Salzburg (form “EK-GZ:09/2018”). All participants signed an informed consent upon enrollment and were informed about their rights, data use, and who to contact in case there are any questions. Participants could terminate their involvement at any time without providing a reason. The data were processed exclusively by project staff and must not be disclosed to third parties. All persons working on the fit4AAL project were subject to confidentiality obligations. Data were collected, stored, and analyzed in accordance with the General Data Protection Regulation (GDPR). Participation in the waiting-list group was compensated. Participants received a shopping voucher as an incentive for waiting. Furthermore, the study was conducted under consideration of the World Medical Association Declaration of Helsinki.

## Results

### Overview

**Figure 4.**
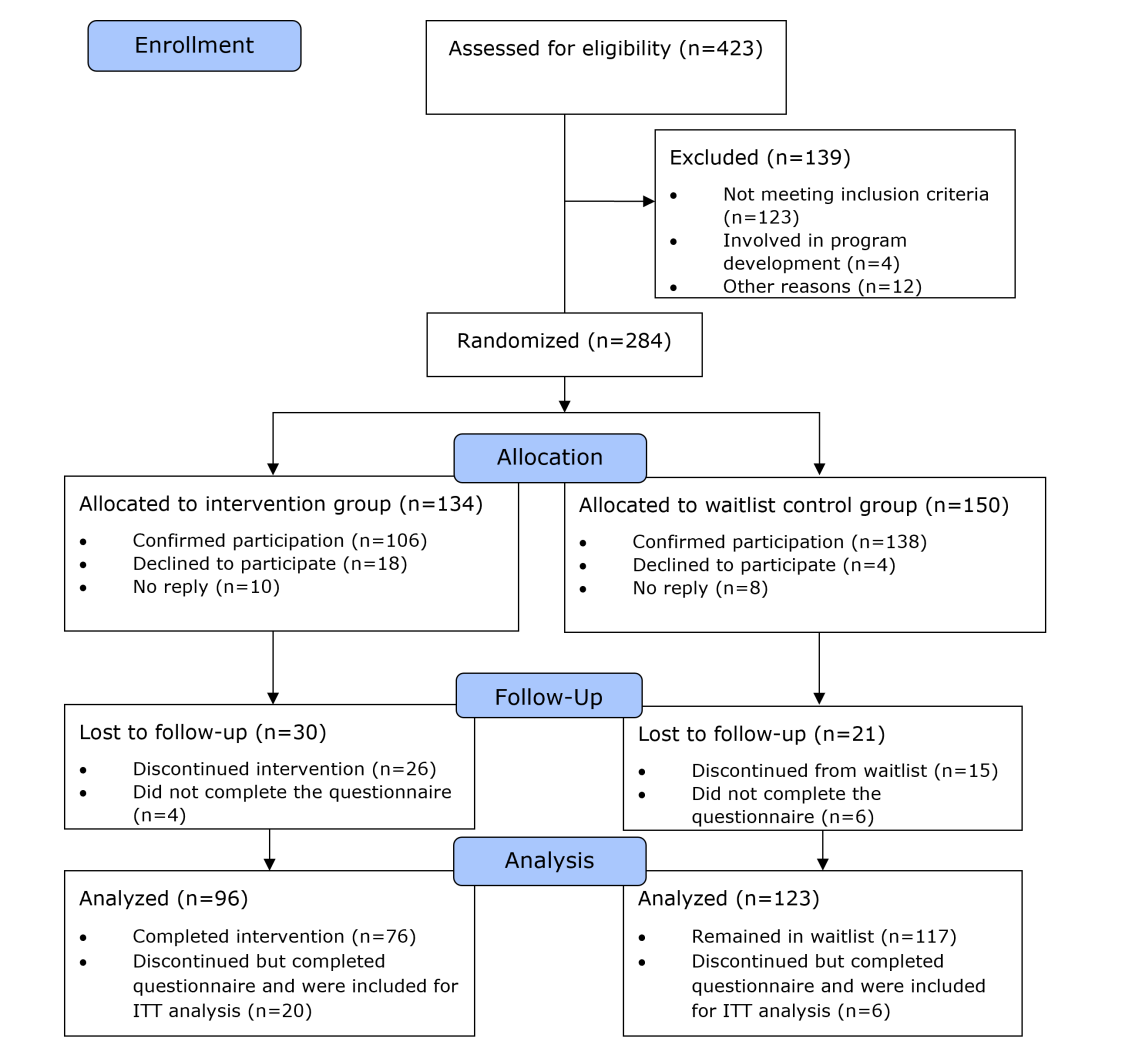
Participant flow adjusted to the CONSORT (Consolidated Standards of Reporting Trials) flow chart. ITT: intention-to-treat.

Figure 4 provides information on the participant flow. In total, 423 people subscribed to the study and were assessed for eligibility. Of the total, 139 had to be excluded. Furthermore, 284 participants were randomly allocated to IG (134 participants) and to the waitlist CG (150 participants). After 8 weeks, the intervention period started, with 106 participants in the IG and 138 in the CG. Participants discontinued for different reasons, such as health issues, lack of time, or lack of interest. In total, 193 participants completed the intervention period, with 76 participants in the IG and 117 in the waitlist CG. In addition, data from 26 participants who discontinued intervention or waitlist were collected. Total sample size for the analysis was 219, with 96 in the IG and 123 in the waitlist CG indicating sufficient statistical power.

### Sample Description

Table 1 summarizes the sample descriptive data at baseline. Participant characteristics were equally distributed between IG and CG. The study samples consisted of rather highly educated adults between 60 and 72 years. About 60% (57/96 and 78/123) of participants in each group reported being physically active at least 1‐2 times a week and rated their health status on average in the upper half.

**Table 1.** Baseline characteristics of the participants (source: own calculations).

Characteristic	Intervention group (n=96)^a^	Control group (n=123)	*P* value
Age (y)			.82
Mean (SD)	65.5 (2.5)	65.4 (2.3)	
Minimum-maximum	60-72	62-71	
Sex, n (%)			.77
Female	75 (78.0)	94 (76.4)	
Male	21 (22.0)	29 (23.6)	
Education (ISCED^b^), n (%)			.58
Lower secondary (0‐2)	14 (15.0)	23 (18.7)	
Upper secondary (3)	24 (25.0)	20 (16.3)	
Postsecondary (4)	26 (27.0)	34 (27.6)	
Short-cycle tertiary (5)	9 (9.0)	14 (11.4)	
Bachelor’s degree or higher (6-8)	22 (23.0)	30 (24.4)	
Unknown or missing	1 (1.0)	2 (1.6)	
Region, n (%)			.45
Vienna	45 (47.0)	68 (52.7)	
Salzburg	51 (53.0)	61 (47.3)	
Household size			.80
Mean (SD)	1.8 (0.6)	1.9 (0.8)	
Minimum-maximum	1-4	1-5	
Physically active, n (%)			.05
Never	9 (9.0)	27 (22)	
Less than once a week	16 (17.0)	18 (14.6)	
1‐2 times a week	52 (54.0)	50 (40.7)	
3‐4 times a week	19 (20.0)	28 (22.8)	
Health status			.29
Mean (SD)	3.3 (0.6)	3.1 (0.6)	
Minimum-maximum	2-5	1-5	

a Numbers may not sum up to 100% due to rounding.

b ISCED: International Standard Classification of Education.

### Outcome Indicators at Baseline

Table 2 shows the initial levels for each investigated physical functioning item before intervention. The numbers show that more than 60% of each group chose a response referring to rate an activity at least as “easy.” The first indicator, “climb up stairs and carry something,” was mostly assessed as being “easy” equal to “lift and carry groceries.” “Walk 1 km” and “stretching to the toes” were rated mostly as “very easy.” All 4 items contained outcome levels with less than 5% of all responses. Thus, we collapsed the affected outcome levels. Consequently, estimated treatment effects refer to the collapsed outcome levels.

**Table 2.** Outcome measures at baseline (source: own calculations).

Activity and response	Intervention group (n=96), n (%)^a^	Control group (n=123), n (%)	*P* value
Climb up stairs and carry something			.34
Difficult	10 (11.0)	16 (13.7)	
Somewhat difficult	10 (11.0)	11 (9.4)	
Somewhat easy	17 (18.0)	31 (26.5)	
Easy	42 (44.0)	37 (31.6)	
Very easy	16 (17.0)	22 (18.8)	
Lift and carry groceries			.08
Difficult and somewhat difficult	13 (14.0)	20 (16.8)	
Somewhat easy	11 (12.0)	29 (24.4)	
Easy	44 (47.0)	43 (36.1)	
Very easy	26 (28.0)	27 (22.7)	
Walk 1 km			.09
Difficult and somewhat difficult	7 (7.0)	17 (13.8)	
Somewhat easy	7 (7.0)	15 (12.2)	
Easy	22 (23.0)	34 (27.6)	
Very easy	60 (63.0)	57 (46.3)	
Stretch to the toes			.67
Difficult and somewhat difficult	14 (15.0)	19 (15.5)	
Somewhat easy	8 (8.0)	14 (11.4)	
Easy	30 (31.0)	30 (24.4)	
Very easy	44 (46.0)	60 (48.8)	

a Numbers may not sum up to 100% due to rounding.

### Model Results

#### Climb Up Stairs and Carry Something

The app-based physical activity intervention significantly increased the self-perceived ability to climb up stairs and carry something (odds ratio [OR] 2.67, 95% CI 1.37-5.18; *P*=.004, hypothesis 1 confirmed; Table 3).

**Table 3.** Results of the ordered logit mixed models (source: own calculations).

Variables	Climb up stairs and carry something (n=620)			Lift and carry groceries (n=626)		Walk 1 km (n=642)		Stretch to the toes (n=645)	
	OR^a^ (95% CI)		*P* value	OR (95% CI)	*P* value	95% CI	*P* value	95% CI	*P* value
Treatment effect	2.67 (1.37 to 5.18)		.004	1.99 (1.02 to 3.89)	.04	1.91 (0.85 to 4.30)	.12	1.31 (0.62 to 2.76)	.48
Intervention period (reference preintervention)									
Postintervention	1.98 (0.98 to 3.96)		.06	1.12 (0.52 to 2.44)	.77	1.31 (0.59 to 2.91)	.51	0.98 (0.42 to 2.28)	.97
Group (reference control group)									
Intervention group	1.06 (0.50 to 2.25)		.88	1.39 (0.67 to 2.87)	.38	0.53 (0.66 to 3.57)	.32	0.73 (0.29 to 1.88)	.52
Time	0.63 (0.42 to 0.94)		.02	0.82 (0.54 to 1.25)	.36	0.75 (0.47 to 1.18)	.22	0.94 (0.59 to 1.52)	.81
Health	6.49 (3.04 to 13.85)		<.001	5.52 (3.08 to 9.87)	<.001	10.57 (5.05 to 22.11)	<.001	5.94 (2.69 to 13.33)	<.001
Age	0.83 (0.66 to 1.04)		.10	0.75 (0.61 to 0.92)	.006	0.69 (0.54 to 0.88)	.003	0.97 (0.75 to 1.25)	.80
Sex (reference male)									
Female	0.36 (0.10 to 1.33)		.12	0.29 (0.09 to 0.95)	.04	0.27 (0.07 to 1.07)	.06	17.16 (3.58 to 82.14)	<.001
Education (reference lower secondary)									
Upper secondary	2.28 (0.68 to 7.66)		.18	2.42 (0.73 to 7.98)	.15	2.21 (0.56 to 8.79)	.26	2.07 (0.46 to 9.44)	.35
Post-secondary	3.00 (0.92 to 9.81)		.07	2.59 (0.80 to 8.39)	.11	2.71 (0.80 to 9.18)	.11	1.71 (0.42 to 6.99)	.46
Short-cycle tertiary	1.21 (0.38 to 3.90)		.75	1.84 (0.50 to 6.80)	.36	1.92 (0.44 to 8.47)	.39	0.61 (0.09 to 4.16)	.62
Bachelor’s degree or higher	4.35 (1.40 to 13.50)		.01	2.65 (0.85 to 8.25)	.09	6.09 (1.74 to 21.23)	.005	1.60 (0.39 to 6.59)	.52
Physically active (reference never)									
Less than once a week	1.84 (0.42 to 8.07)		.42	1.10 (0.31 to 3.91)	.89	1.71 (0.42 to 7.03)	.46	0.51 (0.09 to 2.91)	.45
1‐2 times a week	3.77 (1.03 to 13.77)		.04	0.74 (0.29 to 1.88)	.53	5.47 (1.61 to 18.66)	.007	1.94 (0.45 to 8.36)	.37
3‐4 times a week	4.87 (1.07 to 22.09)		.04	1.65 (0.50 to 5.43)	.41	15.57 (3.44 to 70.49)	<.001	3.40 (0.70 to 16.50)	.13
Region (reference Vienna)									
Salzburg	2.39 (1.03 to 5.32)		.03	0.95 (0.47 to 1.96)	.90	0.74 (0.31 to 1.75)	.49	1.07 (0.39 to 2.92)	.90
Household size	1.28 (0.76 to 2.17)		.35	1.00 (0.64 to 1.58)	.99	0.47 (0.27 to 0.83)	.01	0.72 (0.37 to 1.42)	.34
Random intercept	5.74 (1.07 to 5.32)		—^b^	4.58 (3.14 to 6.68)	—	10.62 (6.94 to 16.28)	—	6.17 (3.89 to 9.78)	—
Cutpoint 1	−8.75 (−24.77 to 7.27)		—	−16.69 (−31.37 to −2.01)	—	1.21 (−17.22 to 19.64)	—	(−38.66 to −4.47)	—
Cutpoint 2	−6.77 (−22.75 to 9.22)		—	−14.81 (−29.46 to −0.16)	—	3.08 (−15.36 to 21.52)	—	−19.69 (−36.76 to −2.63)	—
Cutpoint 3	−4.76 (−20.74 to 11.23)		—	−11.56 (−26.17 to 3.06)	—	6.1 (−12.38 to 24.59)	—	−16.89 (−33.9 to 0.12)	—
Cutpoint 4	−1.05 (−17.04 to 14.95)		—	—	—	—	—	—	—

a OR: odds ratio.

b Not applicable.

As Figure 3A shows, after the intervention, the probability of rating this activity as “very easy” increased by 8.8 percentage points (95% CI 2.6-14.9; *P*=.005) in the IG compared with the CG, and decreased for “somewhat easy” by 3.4 percentage points (95% CI −5.9 to −0.9; *P*=.008), for “somewhat difficult” by 3.7 percentage points (95% CI −6.4 to −1.0; *P*=.007), and for “difficult” by 3.6 percentage points (95% CI −6.9 to −0.2; *P*=.04). No significant change was estimated for the response category “easy” (95% CI −1.5 to 5.4; *P*=.27).

Predicted probabilities in Table 4 showed that in the IG, the estimated probability for rating the activity as “very easy” increased from 14% to 27.6% and decreased for “somewhat easy” from 22.1% to 17.1%, for “somewhat difficult” from 15.6% to 9.2%, and for “difficult” from 12.3% to 5.1%. At the same time, in the CG, the probability for “very easy” increased to a lesser extent from 13.6% to 18.5%, and decreased for “somewhat easy” from 22.2% to 20.6%, for “somewhat difficult” from 15.8% to 13.1%, and for “difficult” from 12.6% to 8.9%.

**Table 4.** Average marginal effects and predicted probabilities for each outcome for intervention group and control group (source: own calculations).

Item and outcome level	Intervention group (n=96)		Control group (n=123)		Treatment effect, percentage points (95% CI)	*P* value
	Pre in %	Post in %	Pre in %	Post in %		
Climb up stairs and carry something						
Difficult	12.3	5.1	12.6	8.9	−3.6 (–6.9 to –0.2)	.04
Somewhat difficult	15.6	9.2	15.8	13.1	−3.7 (–6.4 to –1.0)	.007
Somewhat easy	22.1	17.1	22.2	20.6	−3.4 (–5.9 to –0.9)	.008
Easy	36	41.1	35.7	38.8	1.9 (–1.5 to 5.4)	.27
Very easy	14	27.6	13.6	18.5	8.8 (2.6 to 14.9)	.005
Lift and carry groceries						
Difficult and somewhat difficult	15.5	10	18.1	17.1	−4.5 (–9.7 to 0.8)	.10
Somewhat easy	18.9	15.3	20.4	19.9	−3.2 (–6.3 to –0.1)	.04
Easy	40.3	40.2	39.8	39.5	−0.2 (–3.2 to 2.7)	.88
Very easy	25.3	34.4	22	23.2	7.9 (0.5 to 15.3)	.04
Walk 1 km						
Difficult and somewhat difficult	10.1	6.4	12.2	10.8	2.2 (5.8 to 1.3)	.22
Somewhat easy	11.7	8.8	13.1	12.2	−2.0 (−4.6 to –0.5)	.11
Easy	27	23.8	28.1	27.4	−2.6 (−5.6 to –0.4)	.09
Very easy	51.2	60.9	46.6	49.5	6.8 (−1.6 to 15.3)	.11
Stretch to the toes						
Difficult and somewhat difficult	15.3	14	13.6	13.8	−1.4 (−5.4 to 2.5)	.47
Somewhat easy	12.9	12.2	12.1	12.1	−0.7 (−2.5 to 1.2)	.49
Easy	27.4	26.9	26.9	26.8	−0.5 (−2.0 to 1.0)	.52
Very easy	44.4	46.8	47.4	47.2	2.6 (−4.7 to 9.9)	.48

#### Lift and Carry Groceries

We found a significant effect of the intervention on the self-perceived ability to lift and carry groceries (OR 1.99, 95% CI 1.02-3.89; *P*=.04, hypothesis 2 confirmed; Table 3).

Figure 3B illustrates that rating this activity as “very easy” increased by 7.9 percentage points (95% CI 0.5-15.3; *P*=.04) and as “somewhat easy” decreased by 3.2 percentage points (95% CI −6.3 to −0.1; *P*=.04) in the IG compared with the CG. The average marginal effects did not significantly differ for the outcome levels “easy” (95% CI −3.2 to 2.7; *P*=.88) and “somewhat difficult” (95% CI −9.7 to 0.8; *P*=.10).

Table 4 shows that the category with the highest predicted number of participants was “easy” with about 40% before and after the intervention in both groups. Response category “very easy” increased from 25.3% to 34.4% in the IG and remained almost constant (from 22% to 23.2%) in the CG, while for “somewhat easy” it decreased from 18.9% to 15.3% (IG) and from 20.4% to 19.9% (CG), and for “somewhat difficult” from 15.5% to 10% and from 18.1% to 17.1% in the IG and in the CG, respectively.

#### Walk 1 km

For the ability to walk 1 km, neither a significant increase nor the prevention of a decline for the IG could be found (OR 1.91, 95% CI 0.85-4.30; *P*=.12, hypothesis 3 rejected; Table 3). While the share of participants choosing the outcome level “very easy” increased from 51.2% to 60.9% in the IG and from 46.6% to 49.5% in the CG, the share of participants indicating to be able to perform this activity in an “easy,” “somewhat easy,” and “somewhat difficult” way decreased as shown in Table 4.

#### Stretch to the Toes

The intervention did not significantly improve the self-perceived ability to stretch to the toes (OR 1.31, 95% CI 0.62-2.76; *P*=.48, hypothesis 4 rejected; Table 3) as predicted probabilities hardly changed for any response category (Figure 3D). It was estimated that the number of participants in both groups who chose “very easy” was about 47% and for “easy” about 27% before and after the intervention as reported in Table 4. Furthermore, “somewhat easy” and “somewhat difficult” remained at estimated probabilities of 12% to 15% in both groups.

## Discussion

### Principal Findings

This study investigated the impact of an app-based physical activity program on the perception of physical functioning in older adults in their early years of retirement. Results showed that the multicomponent fitness exercise program was effective in terms of significantly improving the perception of physical functioning for some activities but not for all. The common daily activities of “climbing up stairs and carrying something” and “lifting and carrying groceries” that refer to the physical abilities of strength, endurance, and balance were significantly improved by the app-based physical activity program. On the contrary, “walk 1 km” and “stretch to the toes” associated mainly with endurance and flexibility, respectively, were not significantly affected by the app-based intervention. Self-perceived abilities related to flexibility and mobility could not be changed; although, the app-based program was developed to offer exercises to support a range of physical functionality including these abilities.

Previous research using objective indicators for assessing the same app also found a significant increase in strength for the subgroup of female participants [25]. The program’s positive impact on objectively measured flexibility for the subgroup of women [25], however, could not be confirmed for the full sample—comprising women and men—when using participants' perceptions on their ability to “stretch to the toes.” This supports the usefulness of using both objective and subjective indicators for gathering detailed information on the effectiveness of interventions.

The results of the app-based fitness program—which supported strength and endurance-related functionalities but not those related to flexibility—could be influenced by the design of the program or the preferences of the participants. We cannot rule out that participants were more likely to complete exercises supporting strength than flexibility. Flexibility exercises were part of the warm-up and cool-down parts of the fitness program, which may be more likely to be skipped than the main training sections. Future research is thus encouraged to combine quantitative and qualitative evaluation methods, including detailed usage data analysis, to gain deeper insights into the reasons for the effects of such programs.

This analysis adds evidence that the app-based multicomponent fitness exercise program is not only able to increase physical activity levels [17] and to enhance strength, endurance, and balance [23,62], but also to improve self-perceived physical functioning which supports maintaining physical independence. Physical independence has been denoted as “the ultimate behavioral goal’” by Rikli and Jones [28]. In the context of “successful aging” and “aging in place,” digital technologies can thus not only be considered as facilitating and monitoring technologies, but also as a preventive measure against functional decline to preserve a good quality of life in old age and enable aging in one’s own home.

We expected the app-based fitness program to either increase the participants’ abilities or at least to prevent a decline. The program contributed to the improvement of some self-assessed competencies. We did not observe preventing a decline in competences in the IG, only improvements. The main reason might be that all participants, in the IG and in the CG, reported a high initial level of physical functioning and a decline in the early years of retirement might be less likely compared with older adults who have been retired for a longer period. However, the improvement of the 2 abilities implies a postponement of physical dependence.

Using self-reported physical functioning, items delivered concrete insights into the perception to cope with daily life situations as highlighted by Seidel et al [29]. In addition, self-reported outcome measures include participants’ belief in self-efficacy, for which it is necessary that people engage in certain activities [30]. Our results indicate a positive impact of the app-based physical activity program on self-efficacy that is mentioned to be of specific importance for older adults to engage in physical activity and to prevent functional limitations [33].

Further strengths of the study refer to the novel app-based physical activity program investigated by a high-quality study design, its implementation, and the high compliance by the participants. The impact of the intervention was investigated by a waitlist randomized controlled trial with 219 participants, with sufficient power and a sample larger than in most fitness app intervention studies [17].

This study has some limitations. First, the study sample had an initial high level of physical functioning, which could lead to ceiling effects. We observed that physically active older people selected themselves into the study, which implies that the study results hold for such a group and people who are a priori interested in such programs. Thus, external validity is limited as the population of people who have recently retired is expected to be more diverse, with older adults being less physically active, implying a higher potential to benefit from the intervention. On the other hand, a more diverse population may also include people who are not interested in physical activity interventions leading to a higher dropout rate. Future studies could aim to also involve people with more diverse levels of physical activities and might also investigate which behavior change strategies are necessary and most effective in convincing people to increase their physical activity levels to improve physical functioning. Second, waitlist control designs may impact results [63]. We cannot rule out that inflating effects affected our study. However, the experiences of the fitness trainers do not point to an affected behavior of the CG, for example, skipping physical activities while waiting for access to the app-based program. Thus, to the best of our knowledge, the state-of-the-art study design and its implementation resulted in good internal validity. Third, fitness coaching was offered for the IG and CG. Thus, our study results hold for app-based interventions introduced by a fitness coach and combined with a few fitness coach appointments. Finally, the intervention consisted of many components; thus, we cannot single out which of the components was particularly effective.

### Conclusion

The app-based multicomponent fitness program effectively improved self-perceived physical functioning in early retirees, although not all aspects relevant to independent living were enhanced. Future programs should also focus on flexibility and mobility to better support independence. Self-reported outcomes provided insights into self-efficacy. ICT-based prevention programs tailored for older adults could help maintain physical functionality and potentially reduce future health care costs.

## Supplementary material

10.2196/64922 Multimedia Appendix 1 Screenshot of Scio use.

10.2196/64922 Checklist 1 CONSORT (Consolidated Standards of Reporting Trials) checklist.

## Abbreviations

CG: control group
CONSORT-EHEALTH: Consolidated Standards of Reporting Trials of Electronic and Mobile Health Applications and Online Telehealth
CPF: composite physical function
GDPR: General Data Protection Regulation
ICT: information and communications technology
IG: intervention group
ISCED: International Standard Classification of Education
OR: odds ratio

## References

[1] Fusco O, Ferrini A, Santoro M, Lo Monaco MR, Gambassi G, Cesari M (2012). Physical function and perceived quality of life in older persons. Aging Clin Exp Res.

[2] Prasad L, Fredrick J, Aruna R (2021). The relationship between physical performance and quality of life and the level of physical activity among the elderly. J Educ Health Promot.

[3] Kim K il, Gollamudi SS, Steinhubl S (2017). Digital technology to enable aging in place. Exp Gerontol.

[4] Henchoz Y, Meylan L, Goy R (2015). Domains of importance to the quality of life of older people from two Swiss regions. Age Ageing.

[5] Scales K (2021). It is time to resolve the direct care workforce crisis in long-term care. Gerontologist.

[6] Anton SD, Woods AJ, Ashizawa T (2015). Successful aging: advancing the science of physical independence in older adults. Ageing Res Rev.

[7] Fernández-Ballesteros R, Benetos A, Robine JM (2019). The Cambridge Handbook of Successful Aging.

[8] Wiles JL, Leibing A, Guberman N, Reeve J, Allen RES (2012). The meaning of “aging in place” to older people. Gerontologist.

[9] Ollevier A, Aguiar G, Palomino M, Simpelaere IS (2020). How can technology support ageing in place in healthy older adults? A systematic review. Public Health Rev.

[10] Annele U, Satu KJ, Timo ES (2019). Definitions of successful ageing: a brief review of a multidimensional concept. Acta Bio Medica: Atenei Parmensis.

[11] Saadeh M, Xia X, Verspoor E (2023). Trajectories of physical function and behavioral, psychological, and social well-being in a cohort of Swedish older adults. Innov Aging.

[12] Pinto-Bruno ÁC, García-Casal JA, Csipke E, Jenaro-Río C, Franco-Martín M (2017). ICT-based applications to improve social health and social participation in older adults with dementia. A systematic literature review. Aging Ment Health.

[13] McGarrigle L, Boulton E, Todd C (2020). Map the apps: a rapid review of digital approaches to support the engagement of older adults in strength and balance exercises. BMC Geriatr.

[14] Marques EA, Baptista F, Santos DA, Silva AM, Mota J, Sardinha LB (2014). Risk for losing physical independence in older adults: the role of sedentary time, light, and moderate to vigorous physical activity. Maturitas.

[15] Emberson MA, Lalande A, Wang D, McDonough DJ, Liu W, Gao Z (2021). Effectiveness of smartphone-based physical activity interventions on individuals’ health outcomes: a systematic review. Biomed Res Int.

[16] Schweitzer J, Synowiec C (2012). The economics of eHealth and mHealth. J Health Commun.

[17] Muellmann S, Forberger S, Moellers T, Broering E, Zeeb H, Pischke CR (2018). Effectiveness of eHealth interventions for the promotion of physical activity in older adults: a systematic review. Prev Med.

[18] Yerrakalva D, Yerrakalva D, Hajna S, Griffin S (2019). Effects of mobile health app interventions on sedentary time, physical activity, and fitness in older adults: systematic review and meta-analysis. J Med Internet Res.

[19] McGarrigle L, Todd C (2020). Promotion of physical activity in older people using mHealth and eHealth technologies: rapid review of reviews. J Med Internet Res.

[20] Laranjo L, Ding D, Heleno B (2021). Do smartphone applications and activity trackers increase physical activity in adults? Systematic review, meta-analysis and metaregression. Br J Sports Med.

[21] Granet J, Peyrusqué E, Ruiz F (2023). Web-based physical activity interventions are feasible and beneficial solutions to prevent physical and mental health declines in community-dwelling older adults during isolation periods. J Gerontol A Biol Sci Med Sci.

[22] Yi D, Yim J (2021). Remote home-based exercise program to improve the mental state, balance, and physical function and prevent falls in adults aged 65 years and older during the COVID-19 pandemic in Seoul, Korea. Med Sci Monit.

[23] Kim DR, Song S, Kim GM (2021). Effects of ICT-based multicomponent program on body composition and cognitive function in older adults: a randomized controlled clinical study. Clin Interv Aging.

[24] van Het Reve E, Silveira P, Daniel F, Casati F, de Bruin ED (2014). Tablet-based strength-balance training to motivate and improve adherence to exercise in independently living older people: part 2 of a phase II preclinical exploratory trial. J Med Internet Res.

[25] Jungreitmayr S, Kranzinger C, Venek V, Ring-Dimitriou S (2022). Effects of an app-based physical exercise program on selected parameters of physical fitness of females in retirement: a randomized controlled trial. Front Physiol.

[26] Taylor AM, Phillips K, Patel KV (2016). Assessment of physical function and participation in chronic pain clinical trials: IMMPACT/OMERACT recommendations. Pain.

[27] Guralnik JM, Ferrucci L, Pieper CF (2000). Lower extremity function and subsequent disability: consistency across studies, predictive models, and value of gait speed alone compared with the short physical performance battery. J Gerontol A Biol Sci Med Sci.

[28] Rikli RE, Jones CJ (2013). Development and validation of criterion-referenced clinically relevant fitness standards for maintaining physical independence in later years. Gerontologist.

[29] Seidel D, Brayne C, Jagger C (2011). Limitations in physical functioning among older people as a predictor of subsequent disability in instrumental activities of daily living. Age Ageing.

[30] Bandura A, Freeman WH, Lightsey R (1999). Self-Efficacy: The Exercise of Control.

[31] Bandura A (2012). Handbook of Principles of Organizational Behavior: Indispensable Knowledge for Evidence‐based Management.

[32] Neupert SD, Lachman ME, Whitbourne SB (2009). Exercise self-efficacy and control beliefs: effects on exercise behavior after an exercise intervention for older adults. J Aging Phys Act.

[33] McAuley E, Szabo A, Gothe N, Olson EA (2011). Self-efficacy: implications for physical activity, function, and functional limitations in older adults. Am J Lifestyle Med.

[34] Luszczynska A, Schwarzer R (2015). Social cognitive theory. Fac Health Sci Publ.

[35] Ceasar JN, Claudel SE, Andrews MR (2019). Community engagement in the development of an mHealth-enabled physical activity and cardiovascular health intervention (Step It Up): pilot focus group study. JMIR Form Res.

[36] Schneider C, Venek V, Rieser H, Jungreitmayr S, Trukeschitz B, Ring-Dimitriou S, Dimitriou M (2022). Aktives Altern im digitalen Zeitalter: Informations-Kommunikations-Technologie verstehen, nutzen und integrieren.

[37] Jones CJ, Rikli RE (2002). Measuring functional fitness of older adults. J Act Aging.

[38] StataCorp (2017). Stata statistical software: release 15.

[39] Schmitz C (2012). LimeSurvey: an open source survey tool.

[40] Faul F, Erdfelder E, Lang AG, Buchner A (2007). G*Power 3: a flexible statistical power analysis program for the social, behavioral, and biomedical sciences. Behav Res Methods.

[41] Cohen J (1988). Statistical Power Analysis for the Behavioral Sciences.

[42] Chase JAD (2013). Methodological challenges in physical activity research with older adults. West J Nurs Res.

[43] Harrington CN, Wilcox L, Connelly K, Rogers W, Sanford J (2018). Designing health and fitness apps with older adults: examining the value of experience-based co-design.

[44] Trukeschitz B, Blüher M, Michel L, Eisenberg S, Jungreitmayr S, Schechinger M Das app-basierte Bewegungsprogramm „Fit-mit-ILSE: Nutzungserfahrungen: Erkenntnisse aus dem ersten Feldtest des AAL-Projekts „fit4AAL“. Forschungsberichte des WU Forschungsinstituts für Altersökonomie no. 3/2020.

[45] Outdooractive.

[46] Balza JS, Cusatis R, McDonnell SM, Basir MA, Flynn KE (2022). Effective questionnaire design: how to use cognitive interviews to refine questionnaire items. J Neonatal Perinatal Med.

[47] Cuenca-Garcia M, Marin-Jimenez N, Perez-Bey A (2022). Reliability of field-based fitness tests in adults: a systematic review. Sports Med.

[48] Leis KS, Reeder BA, Chad KE, Spink KS, Fisher KL, Bruner BG (2010). The relationship of chronic disease and demographic variables to physical activity in a sample of women aged 65 to 79 years. Women Health.

[49] McKee G, Kearney PM, Kenny RA (2015). The factors associated with self-reported physical activity in older adults living in the community. Age Ageing.

[50] Biernat E, Tomaszewski P (2011). Socio-demographic and leisure activity determinants of physical activity of working Warsaw residents aged 60 to 69 years. J Hum Kinet.

[51] Franco MR, Tong A, Howard K (2015). Older people’s perspectives on participation in physical activity: a systematic review and thematic synthesis of qualitative literature. Br J Sports Med.

[52] King WC, Brach JS, Belle S, Killingsworth R, Fenton M, Kriska AM (2003). The relationship between convenience of destinations and walking levels in older women. Am J Health Promot.

[53] Jenkins ND, Hoogendijk EO, Armstrong JJ (2022). Trajectories of frailty with aging: coordinated analysis of five longitudinal studies. Innov Aging.

[54] Bullinger M, Kirchberger I, Ware J (1995). Der deutsche SF-36 Health Survey Übersetzung und psychometrische Testung eines krankheitsübergreifenden Instruments zur Erfassung der gesundheitsbezogenen Lebensqualität [Article in German Language]. J Public Health.

[55] Altman DG, Doré CJ (1990). Randomisation and baseline comparisons in clinical trials. The Lancet.

[56] Twisk J, Bosman L, Hoekstra T, Rijnhart J, Welten M, Heymans M (2018). Different ways to estimate treatment effects in randomised controlled trials. Contemp Clin Trials Commun.

[57] Senn S (2013). Seven myths of randomisation in clinical trials. Stat Med.

[58] Williams R (2012). Using the margins command to estimate and interpret adjusted predictions and marginal effects. The Stata Journal: Promoting communications on statistics and Stata.

[59] Li C (2013). Little’s test of missing completely at random. The Stata Journal: Promoting communications on statistics and Stata.

[60] Murad H, Fleischman A, Sadetzki S, Geyer O, Freedman LS (2003). Small samples and ordered logistic regression: does it help to collapse categories of outcome?. Am Stat.

[61] StataCorp (2021). Stata statistical software: release 17.

[62] Valenzuela PL, Saco-Ledo G, Morales JS (2023). Effects of physical exercise on physical function in older adults in residential care: a systematic review and network meta-analysis of randomised controlled trials. Lancet Healthy Longev.

[63] Laws KR, Pellegrini L, Reid JE, Drummond LM, Fineberg NA (2022). The inflating impact of waiting-list controls on effect size estimates. Front Psychiatry.

