# Supplementary figures and images for: The Effectiveness of an App-Based Fitness Program on Self-Perceived Physical Functioning in Older Adults: Randomized Waitlist-Controlled Trial
Source: J Med Internet Res. 2025 Aug 18;27:e64922. doi: 10.2196/64922 (PMC12360723; doi:10.2196/64922)

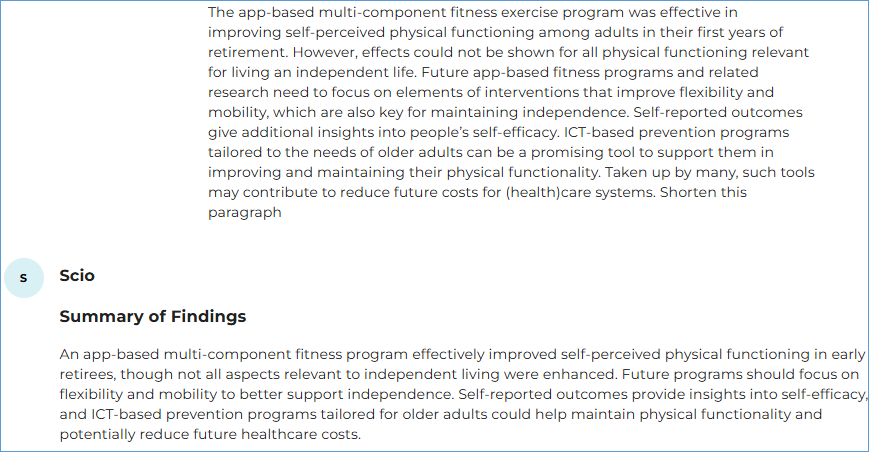

Supplement: Multimedia Appendix 1 [file jmir-v27-e64922-s001.png]
